# Sodium Ethylate

**Published:** 1889-07

**Authors:** George H. Clark

**Affiliations:** Germantown; Clinical Bureau, I. H. A.


### SODIUM ETHYLATE.

GEORGE H. CLARK, M. D., GERMANTOWN.

[Clinical Bureau, I. H. A.]

The treatment of nævi and other vascular tumors by the knife, the cautery, electrolysis, and the common caustics is always unsatisfactory.

Aside from the pain caused, there is usually a more or less unsightly scar left.

It is desirable, particularly where such blemishes are found on the face and other visible parts, that these unsightly defects should be so treated as to cause a minimum of pain, with as little scarring as possible.

To Dr. Benjamin Ward Richardson, of London, we are indebted for two substances that meet these demands: Ethylate of Sodium and Potassium Ethylate.

These substances are prepared as follows: Ethylate of Sodium, or Sodium Alcohol, is made by treating absolute alcohol with pure metallic sodium. Put one-half ounce rectified alcohol into a two-ounce test-tube, set up in a bath of cold water, then add small pieces of pure metallic sodium. Hydrogen will at once escape. Add sodium until the gas ceases to escape, then warm in a bath of one hundred degrees, and add a little more sodium until the gas again ceases to escape, then cool down to fifty degrees and add one-half ounce alcohol. It can be made more active by adding more sodium.

Dr. Richardson says: "I find it good to increase the temperature gradually as the action declines. At last there is obtained a thick, nearly white product, which is a saturated solution of sodium alcohol. "From this solution ethylate of sodium crystallizes out in beautiful crystals, which are soluble in pure alcohol.

The composition of sodium ethylate is $\left.\begin{matrix} C_2H_5 \\ Na \end{matrix}\right\} O.$

"When it is brought into contact with water it is decomposed, the sodium becoming oxidized by the oxygen of the water to form sodium hydrate, and the hydrogen of the water going to reconstitute the common or ethylic alcohol.

"The change of ethylic alcohol into sodium ethylate transforms it from an irritant to a caustic. Laid on any parts of the body, the sodium ethylate is comparatively inert, creating no more change than the redness and tingling caused by common alcohol; but so soon as the part to which the substance is applied gives up a little water, the transformation I have described above occurs; caustic soda is produced in contact with the skin in proportion as water is eliminated by the skin, and therefore a gradual destruction of tissue proceeds, which may be so moderated as hardly to be perceptible, or may be so intensified as to destroy almost like a cutting instrument."

Potassium Alcohol, or Potassium Ethylate. This is made in a similar manner to sodium ethylate, viz.: by bringing pure potassium into contact with pure alcohol.

"The action of the potassium is much more energetic than sodium. I prefer to immerse the potassium under the alcohol in a small glass bell, from which there is a tube to allow of the escape of the liberated hydrogen. When saturation is complete a thick and almost colorless fluid is formed, from which the ethylate may be obtained in solid crystalline state. Exposed to water the potassium ethylate is transformed, as is the sodium ethylate, into ethylic alcohol and hydrate of potassium. The composition of the potassium alcohol is $\left.\begin{matrix} C_2H_5 \\ K \end{matrix}\right\} O.$

"The action of this compound on animal tissues, living and dead, is the same as that of the sodium compound, but is more energetic."

My experience is confined to ethylate of sodium. I began using it some eight years ago. The first case to which I applied it was a lady with an aneurism on the nose. The growth was about the size of a pea, and had been gradually increasing in size. The ethylate was applied with a camel's-hair brush. At first no sensation was experienced, but in a few minutes slight burning was felt. In forty-eight hours a light crust had formed. This was allowed to fall off, which occurred in four days.

The ethylate was again applied, and again the crust was allowed to form and fall off. After several such applications the aneurism had disappeared, and there was no mark left to tell that it had ever existed.

Since that I have used it several times, in cases of the same character, and it has always so acted as to leave nothing to be desired.

In some cases it may be necessary to make a slight puncture in the growth in order to have serum exude, and then the sodium will act more quickly.

As has been stated above, the potassium ethylate is more active than the sodium. In using potassium a glass rod is necessary, but a camel's-hair pencil or a small brush made of a wooden toothpick answers for using the sodium.

Dr. Richardson's latest experience with the sodium ethylate leads him to recommend it as a specific for the treatment of the ordinary raised circumscribed nævus. It is not applicable to the diffused nævus, commonly called *mother's mark.*

"In treating nævus," says Dr. Richardson, "I first dry the surface with a piece of cotton wool; then with a brush I thoroughly coat the dried surface with the solution. The application causes, always, some effusion and redness, accompanied by a little pain, expressed by those who are old enough to describe it as a burning sensation, like the sting of a bee or a nettle. After a short time there is an exudation of water, in drops, from the red surface, which exudation lasts for a few minutes, and is followed by dryness, and sometimes by pallor or duskiness of appearance. In the course of four or five hours a scab begins to form and continues until there is quite a hard crust, which completely covers the nævus, but through which the soft vascular character of the swelling can be detected. After the first crust is fully formed, I pass through it on the third day a fine needle with cutting edges shaped like an old cataract needle, and with this I break up the vascular surface underneath, and on withdrawing the needle make firm pressure with lint on the upper surface. A large drop or two of blood flows out freely, but further escape is easily controlled by a dossil of lint charged with styptic colloid.

"When the bleeding has quite ceased a drop of the ethylate solution is inoculated into the nævus through the punctured opening; a new layer of it is painted over the crust and the crust left as it was.

"The crust may be left four days more, and if, at that time, the vascular softness still remains under it, it must be treated by puncture and re-injection just as before. When at last the crust feels firm and dry beneath the cure may be considered as complete, and the crust may be left to scale off by itself, leisurely.

"In the treatment of raised nævus by this plan, I have never seen the least untoward symptom of moment, and, although some cases have been rather more tedious than others, there has not been one failure of cure."

From this it will be readily seen that in sodium ethylate we have a mild caustic which is capable of doing what nothing heretofore known can do: mildly and gently remove, without leaving a mark behind, unsightly blemishes that cause serious annoyance and discomfort.

